# Editorial: Hybrid Perovskite Crystals Design, Growth, and Their Photoelectric Properties

**DOI:** 10.3389/fchem.2022.938893

**Published:** 2022-08-09

**Authors:** Xinbo Guo, Zhaolai Chen

**Affiliations:** State Key Laboratory of Crystal Materials, Institute of Crystal Materials, Shandong University, Jinan, China

**Keywords:** perovskite, crystal growth, photodetector, nanocrystal, x-ray detector

The past years have seen the rapid development of halide perovskite solar cells, light emission diodes (LEDs), X-ray detectors, and photodetectors, due to the superior optoelectronic properties of perovskite materials, such as tunable bandgap, long carrier diffusion length, and high carrier mobility. Up to now, polycrystalline perovskite solar cells hold a record efficiency of over 25%, which is comparable with the commercialized Si solar cells. Further improving the optoelectronic devices requires tailoring the crystal growth process which is crucial to reduce the defect density and inhibit carrier recombination. Another way to enhance the device performance is the utilization of perovskite single crystals that possess much better electrical properties than polycrystalline perovskites. The main drawbacks of bulk perovskite single crystals include difficult substrate integration, too large thickness, inefficient carrier collection, low photoluminescence quantum yield (PLQY), etc. To take full advantage of the superior properties of perovskite single crystals, thickness and morphology control are necessary.

In order to fully understand the formation of polycrystalline thin films, Kumar et al. reviewed the fabrication process of polycrystalline thin films and the critical factors influencing the crystal growth. This review described three techniques to optimize the film morphology and device performance: substrate temperature treatment, antisolvent treatment, and cosolvent engineering. They think the key parameter deciding the nucleation and subsequent growth of the nucleated particles is the Gibbs free energy. Through changing substrate temperature to control the volatilization rate of solvent or dripping antisolvent, the grain size and coverage of thin films can be modulated. Apart from this, the solvent boiling point, coordination affinity, and dipole strength also play a significant role in deciding the ultimate film morphology, defect density, and charge transfer resistance. This review is instructive for an understanding of crystallization process of perovskite films, which is beneficial for regulating defect density in the active layer to suppress ion migration and improve material properties.

Perovskite single crystals are ideal candidates for X-Ray detectors due to their large carrier mobility-lifetime product. A key challenge is growing large single crystals with controlled thickness directly on substrates. To this end, Feng et al. reported the growth of methylammonium lead tribromide (MAPbBr_3_) single crystals directly on indium tin oxide (ITO) substrates through inverse temperature crystallization and succeeded in controlling the thickness of the crystal wafer through regulating the distance between solution surface and substrate. When the crystal surface is close to the solution surface, the vertical growth stops while the lateral growth continues, thus resulting in a thickness-controlled, substrate-integrated, and inch-sized single-crystal wafer. The crystal thicknesses can be adjusted from 1 to 3.5 mm. Subsequently, through surface polishing and O_3_ treatment, the surface traps are passivated, leading to X-Ray detectors with a sensitivity of 632 µC Gyair^−1^ cm^−2^ under –5 V bias, which is superior to that of commercial α-Se detectors. This work provides an effective way to fabricate substrate-integrated and large-sized thin perovskite wafers for sensitive X-ray detection, which will promote the real-world application of single crystal X-ray detectors, such as medical imaging.

The most widely investigated perovskite single crystals are based on lead, such as MAPbI_3_ and MAPbBr_3_. However, the absorption range of lead-based perovskite single crystals is relatively narrow, which limits their application in infrared light detection. Learning from polycrystalline perovskites, doping with Sn^2+^ represents an effective way to extend the absorption range of perovskite single crystals into the infrared region. Wu et al. reported the growth of micrometer-thick Pb-Sn mixed single-crystal thin films by combining the inverse temperature crystallization and space-confined strategy. The as-grown 15-μm-thick MAPb_0.5_Sn_0.5_I_3_ single-crystal thin films show red-shifted absorption to 950 nm, which makes them ideal candidates for infrared detection. Under 905 nm light illumination, the as-fabricated photodetectors show a responsivity of 0.514 A/W and a specific detectivity of 1.4974×10^11^ Jones. Moreover, the infrared photodetectors exhibit good operational stability, which can be attributed to the low trap density and good stability of perovskite single crystals. In addition to infrared light detection, an extension of absorption range also benefits photovoltaic applications, which has been widely reported in polycrystalline solar cells. This is the first report about growth of lead-tin mixed single crystals, which can help to investigate the intrinsic properties. However, the oxidation of Sn^2+^ will bring about large amounts of charge traps, causing adverse effects on the material properties and device performance. Therefore, how to inhibit oxidation of Sn^2+^ during crystal growth should be investigated.

Perovskite nanocrystals (NCs), nanometer-scale perovskite single crystals capped with surfactant molecules and dispersed in a non-polar solution, are promising for optoelectronic applications due to their spectral tunability. Hao et al. reviewed the recent development of optoelectronic applications of perovskite NCs, including solar cells, LEDs, and lasers. The black phase of phase-unstable perovskite materials can be stabilized in the form of NCs, such as CsPbI_3_, leading to a high efficiency of 17.39%. The high PLQY of perovskite NCs endows corresponding LEDs with high external quantum efficiency and color purity. At the end of this review, the authors discuss the challenges in this research field and possible solutions. Reducing the dimensionality of single crystals to zero yields endows with their characteristic properties, including near-unity quantum yield, high color purity, and size or surface-chemistry-dependent emission spectra, which broadens their application in light-emitting diodes. To achieve better device performance, the nucleation and growth of perovskite NCs should be separated in time for the synthesis of target nanocrystals with controlled size and high ensemble uniformity. Besides, a deep understanding of how ligands affect nucleation, growth, and shape evolution should be pursued.

